# Peptide receptor radionuclide therapy using radiolabeled somatostatin analogs: focus on future developments

**DOI:** 10.1007/s40336-014-0054-2

**Published:** 2014-03-05

**Authors:** Sander M. Bison, Mark W. Konijnenberg, Marleen Melis, Stefan E. Pool, Monique R. Bernsen, Jaap J. M. Teunissen, Dik J. Kwekkeboom, Marion de Jong

**Affiliations:** 1 Department of Nuclear Medicine, Erasmus MC, Rotterdam, The Netherlands; 2 Department of Radiology, Erasmus MC, Rotterdam, The Netherlands; 3 Department of Medical Informatics, Erasmus MC, Rotterdam, The Netherlands

**Keywords:** Neuroendocrine tumor, Peptide receptor radionuclide therapy, Somatostatin analogs, Dosimetry

## Abstract

Peptide receptor radionuclide therapy (PRRT) has been shown to be an effective treatment for neuroendocrine tumors (NETs) if curative surgery is not an option. A majority of NETs abundantly express somatostatin receptors. Consequently, following administration of somatostatin (SST) analogs labeled with γ-emitting radionuclides, these tumors can be imaged for diagnosis, staging or follow-up purposes. Furthermore, when β-emitting radionuclides are used, radiolabeled peptides (radiopeptides) can also be used for the treatment for NET patients. Even though excellent results have been achieved with PRRT, complete responses are still rare, which means that there is room for improvement. In this review, we highlight some of the directions currently under investigation in pilot clinical studies or in preclinical development to achieve this goal. Although randomized clinical trials are still lacking, early studies have shown that tumor response might be improved by application of other radionuclides, such as α-emitters or radionuclide combinations, or by adjustment of radiopeptide administration routes. Individualized dosimetry and better insight into tumor and normal organ radiation doses may allow adjustment of the amount of administered activity per cycle or the number of treatment cycles, resulting in more personalized treatment schedules. Other options include the application of novel (radiolabeled) SST analogs with improved tumor uptake and radionuclide retention time, or a combination of PRRT with other systemic therapies, such as chemotherapy or treatment with radio sensitizers. Though promising directions appear to bring improvements of PRRT within reach, additional research (including randomized clinical trials) is needed to achieve such improvements.

## Introduction

Neuroendocrine tumors (NETs) are well-differentiated tumors derived from diffuse neuroendocrine cells in the lung, gut or pancreas. NETs are rare, having an incidence of 2–5 per 100,000 inhabitants [1–3]; their prevalence, however, is much higher on account of the relatively slow progression rate of the disease [3]. In general, NETs are diagnosed at a relatively late stage, with metastatic spread present at the time of diagnosis in the majority of patients [3]. Often, therefore, curative surgery is no longer an option. Since chemotherapy and external beam therapy are incapable of treating distant metastases, in most cases these therapeutic options are of limited value [4]. Peptide receptor radionuclide therapy (PRRT) using radiolabeled somatostatin (SST) analogs has proven to be an effective therapeutic option for NET patients with metastasized disease, as it allows targeted delivery of therapeutic radionuclides to tumor cells [5, 6]. Despite the fact that high tumor response rates have been reported after treatment with^177^Lu-DOTA,Tyr^3^-octreotate (DOTA = 1,4,7,10-tetraazacyclododecane-1,4,7,10-tetra-acidic acid) (^177^Lu-DOTATATE) [7] and ^90^Y-DOTA,Tyr^3^-octreotide (^90^Y-DOTATOC) [8], complete responses are still rare, indicating that there is room for improvement of PRRT. The aim of this review is to describe directions that may lead to improvement of imaging and especially treatment of NETs with radiolabeled SST analogs.

SST is a biologically active neuropeptide secreted by the hypothalamus. It acts by binding to G-protein-coupled somatostatin receptors (SSTRs) expressed in different organs in the body, such as the gastrointestinal tract and the pancreas [9]. SST inhibits the secretion of a wide range of hormones. Besides this normal organ expression, SSTRs are (over)expressed by certain malignant tissues, in particular most NETs [10]. SSTRs consist of five G-protein-coupled receptors, subtypes SSTR1–SSTR5 [11], of which SSTR2, in particular, is (over)expressed by NETs [12]. The abundant expression of SSTRs by the majority of NETs enables their visualization in patients. This is achieved, using nuclear imaging techniques, by receptor targeting with radiolabeled SST peptide analogs such as octreotide (D-Phe-c[Cys-Phe-D-Trp-Lys-Thr-Cys]-Thr(ol)) or Tyr^3^-octreotate (D-Phe-c[Cys-Tyr-D-Trp-Lys-Thr-Cys]-Thr) [13, 14]. These stabilized eight-amino acid compounds are derived from native SSTs which consist of 14 or 28 amino acids [15]. Unlabeled SST analogs like long-acting release octreotide (octreotide LAR) are currently applied as initial treatment for patients with metastatic midgut NETs [16]. Octreotide LAR has been shown to have a positive influence on clinical symptoms as well as some tumor-stabilizing effects, leading to a lengthening of time to progression compared with placebo [16].

Functional imaging using SPECT or PET imaging with the radiolabeled SST analogs ^111^Indium-DTPA (diethylenetriaminepentaacetic acid) -octreotide (^111^In-octreotide, or Octreoscan^®^; Mallinckrodt, Petten, the Netherlands) [13], ^68^Ga-DOTA-Tyr^3^-octreotide (^68^Ga-DOTATOC), ^68^Ga -DOTA, 1-Nal^3^-octreotide (^68^Ga -DOTANOC), ^68^Ga-DOTA-Tyr^3^-octreotate (^68^Ga-DOTATATE) [17], ^99m^Tc-EDDA/HYNIC-octreotate [18], or ^99m^Tc-EDDA/HYNIC-octreotide [19] is being widely applied in clinical practice for diagnosis, staging and monitoring of NETs.

^111^In-octreotide is currently the only registered imaging tracer [20]. Over the last few years, however, SST analogs radiolabeled with the positron emitter ^68^Ga have been increasingly used for PET imaging. Compared with SPECT using ^111^In-labeled analogs, PET using ^68^Ga-labeled analogs resulted in a higher spatial resolution, better tissue contrast, and a higher sensitivity for detection of metastases. Several studies have shown PET with ^68^Ga-labeled SST analogs to be superior to SPECT performed using ^111^In-labeled STT analogs [21, 22]. In addition, as ^68^Ga is generator produced [23], it allows for in-house labeling and applications of ^68^Ga in nuclear medicine departments which do not have access to a cyclotron.

Following the successful applications of ^111^In-octreotide for imaging of NETs, the next logical step was to apply this radionuclide, not only emitting γ radiation but also therapeutic Auger and conversion electrons, at high activities for PRRT of metastasized disease as well [24, 25]. Although treatment with ^111^In-octreotide often resulted in symptom relief in patients with metastasized NETs, objective tumor responses were rare, especially in patients with advanced disease and in those with large tumors [8, 24, 25]. Application of ^177^Lu-DOTATATE and ^90^Y-DOTATOC, on the other hand, resulted in impressive therapeutic effects [8, 26–29]. Since ^177^Lu also emits γ rays, ^177^Lu-labeled peptides can be used for treatment as well as for dosimetry and monitoring of tumor response. The first clinical phase III study to evaluate safety and tolerability of ^177^Lu-DOTATATE and compare therapeutic responses after ^177^Lu-DOTATATE with those after treatment with a high dose of the unlabeled SST analog octreotide LAR is currently running in several countries (http://clinicaltrials.gov/ct2/show/NCT01578239?term=NCT01578239&rank=1).

As mentioned above, PRRT has been shown to be a promising treatment option for NET patients. Several excellent reviews have recently described the current status of PRRT in great detail [30, 31]. Within the space constraints of this article we cannot cover every aspect of this exciting field, but we nevertheless aim to help readers appreciate the available options for increasing tumor response after PRRT and to point out some of the latest developments. On the basis of published research, we discuss, below, five ways of increasing the therapeutic effects of PRRT:

1. Recently developed STT analogs acting as receptor antagonists seem to be a highly promising alternative to the receptor agonists currently applied in clinical practice, as several newly developed SSTR antagonists have shown increased tumor uptake compared to STTR agonists [32–34], leading to higher tumor radiation doses. We report on recently achieved results in different tumor models, and discuss the possible mechanisms behind these results and the translation of preclinical studies into the clinic.
2. The use of combinations of selected radionuclides for labeling SST analogs might improve tumor responses. As dose rate, emitted energies and linear energy transfer (LET) are specific for every radionuclide, the radionuclides with the most appropriate characteristics could be combined to obtain optimal effects. Since metastases range in size from small to large tumor masses, we report on the published advantages of combined applications of ^177^Lu and ^90^Y for the treatment of small and large metastases, respectively. Furthermore, we highlight the use of the several most promising α-emitters, which are currently being applied in PRRT in experimental studies.
3. Increased uptake of radionuclides in liver metastases has been achieved after **intra-arterial (i.a.) administration (into the hepatic artery) as opposed to intravenous (i.v.) injection. Below, we describe preclinical and clinical results achieved after i.a. injection and focus on points of interest concerning this new therapeutic approach.
4. Dosimetry during PRRT is of great interest and application of patient-specific dosimetry might allow safe administration of additional treatment cycles to possibly increase tumor response to PRRT.
5. Finally, the combination of PRRT with other therapies might increase the effectiveness of treatment for NET patients. From this perspective, a new application of PRRT is to use it in a neo-adjuvant or adjuvant setting, to allow curative surgery after tumor mass reduction by PRRT or to prevent the development of metastases after spread of tumor cells during surgery. We also focus on increased therapeutic responses after combined PRRT and chemotherapy. Promising combinations of PRRT and chemotherapeutics are under preclinical as well as clinical evaluation.

## Recently developed somatostatin analogs

The SST analogs currently most widely used in the clinical setting include ^111^In- octreotide (Octreoscan^®^) and ^68^Ga-DOTATOC/DOTATATE/DOTANOC for imaging, and ^177^Lu-DOTATATE and ^90^Y-DOTATOC for therapy. Several novel, radiolabeled SST analogs are currently under preclinical and clinical evaluation, as recently reviewed by Fani et al. [30]. Of particular interest are the pansomatostatin analogs, targeting multiple SSTR subtypes [35], and SSTR antagonists. As pansomatostatin analogs like DOTA-lanreotide target more SSTR subtypes than, e.g., DOTATOC, the use of DOTALAN can be considered in patients lacking tumor uptake of DOTATOC [36].

Very promising results have been reported with regard to the application of SSTR antagonists. Until recently it was generally assumed that receptor-targeting ligands should act as receptor agonists to promote efficient internalization of receptor ligand complexes into tumor cells, causing accumulation and long retention of radionuclides within tumors [37]. However, recent studies have shown significantly increased tumor targeting with SSTR antagonists, despite minimal or no internalization of the receptor antagonist complex into tumor cells [32]. Receptor antagonists (e.g.,^111^In-DOTA-SST-ANT) with receptor affinity comparable to that of SSTR agonists readily bind SSTR-expressing tumors to a higher extent than agonists and with a long tumor retention time, as described in an HEK-SSTR2 tumor-bearing mouse study [38]. Factors contributing to this phenomenon include the fact that receptor antagonists occupy more binding sites and show a lower dissociation rate than agonists [32]. Cescato et al. [33] evaluated the in vitro binding of the receptor antagonist ^177^Lu-DOTA-BASS in comparison with that of ^177^Lu-DOTATATE in a study of tissue sections of surgically resected SSTR2-expressing tumor samples. In all cases, the tumor tissues were more intensely labeled using the SSTR antagonist, demonstrating that the antagonistic radioligand detected more binding sites in a large variety of different tumor types, including NETs. On average, 4.2-fold increased binding was found using ^177^Lu-DOTA-BASS. This improved binding may increase the sensitivity of imaging with such receptor antagonist tracers. The first clinical data published thus far comprise a feasibility study in five patients, in whom it was confirmed that ^111^In DOTA-BASS provided a higher tumor uptake and better visualization of metastatic NETs than ^111^In-DTPA-octreotide [34]. Moreover, the kidney retention of the antagonistic compound was lower, resulting in a 5.2 times higher tumor-to-kidney ratio in favor of the receptor antagonist. Also the liver radiation dose appeared to be lower using the receptor antagonists. The lower renal and liver doses, as seen in preclinical and clinical studies [32, 34, 39], can be explained by charge differences between the two compounds.

High tumor uptake, long tumor retention time and less physiological retention of radioactivity in healthy organs indicate that SSTR antagonists are very promising not only for diagnostic, but also for therapeutic purposes. A disadvantage of these antagonists is the fact that their tumor uptake and retention are highly influenced by the choice of chelator and the radionuclide being used [40]. Therefore, it can be difficult to predict tumor dosimetry for PRRT using a diagnostic SSTR antagonist labeled with another radionuclide.

Since SST is a hormone with a repressive effect on tumor growth, SSTR antagonists may theoretically exert a tumor-proliferating effect. However, as yet, there has been no clinical or preclinical report of increased tumor proliferation after treatment with SSTR antagonists. More clinical trials now need to be performed to confirm the safety and effectiveness of applications of these peptide analogs.

## Application of radionuclide combinations and α-emitters

^90^Y and ^177^Lu are currently the most widely applied radionuclides for treatment with radiolabeled SST analogs. The high-energy electrons (11 mm maximum tissue penetration) emitted by ^90^Y suggest that this radionuclide will be more effective in larger tumor masses (optimal diameter of 34 mm [41]) as smaller tumors will not absorb all the energy released. Accordingly, the low-energy electrons emitted by ^177^Lu (1.8 mm maximum tissue penetration) make this radionuclide more suitable for treatment of smaller tumor masses (optimal diameter of 2 mm) [41]. These characteristics suggest that an optimal anti-tumor response in larger tumor masses as well as in smaller metastases could be achieved using a combination of both ^90^Y-DOTATOC and ^177^Lu-DOTATATE. This was confirmed in a preclinical study in rats bearing both smaller and larger tumors, mimicking the varying size of metastases that can be found within a single patient. The combination of ^90^Y-DOTATOC and ^177^Lu-DOTATATE gave superior results compared with a single dose of either ^90^Y-DOTATOC or ^177^Lu-DOTATATE [42]. Reports of the first clinical applications of combinations of both ^90^Y-DOTATOC and ^177^Lu-DOTATATE were published recently. Kunikowska et al. [43] performed a study in patients treated with ^90^Y-DOTATATE alone or ^177^Lu-DOTATATE plus ^90^Y-DOTATATE (concurrent therapy with 1:1 radioactivity ratio). The combined treatment resulted in longer overall survival times than were obtained with ^90^Y-DOTATATE alone, whereas the safety of both methods was comparable. Villard et al. [44] retrospectively compared treatment with alternating sequential ^177^Lu-DOTATOC and ^90^Y-DOTATOC (DUO-PRRT) in 237 patients versus ^90^Y-DOTATOC alone in 249 patients and concluded that their results suggested a longer survival after DUO-PRRT. A prospective clinical study, with a randomized control group and applying patient-specific dosimetry calculations is still lacking, however. As discussed by Savolainen et al. [45], an optimal clinical combination of the two radiopharmaceuticals should be determined on a patient-specific basis. As will be discussed later, the kidneys are among the dose-limiting organs and considering the substantially lower dose rate to the kidneys of ^177^Lu compared with ^90^Y, the biologically effective dose (BED) to the kidneys should be calculated for the specific tandem combination being applied.

**Fig. 1:**
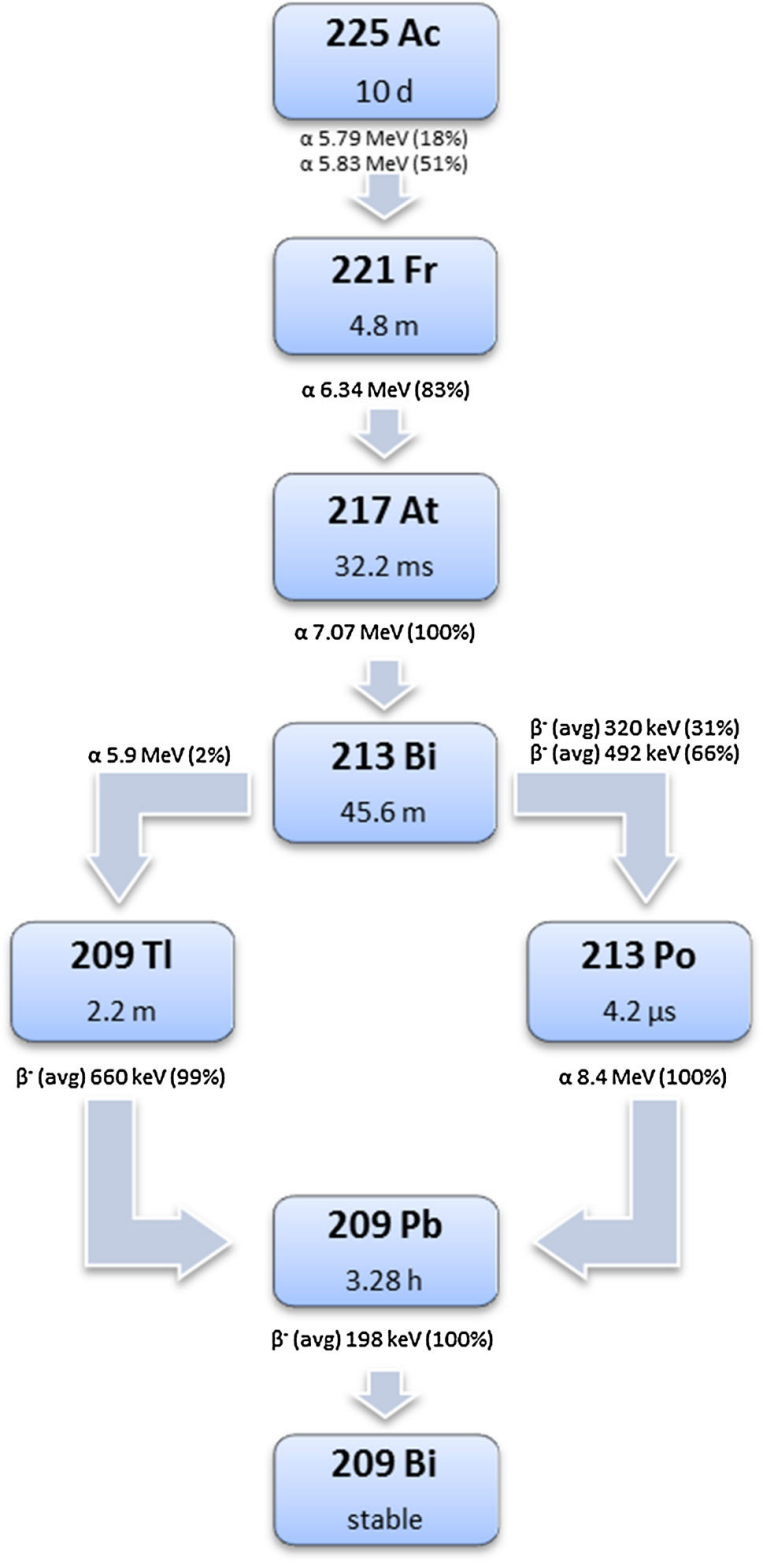
Decay of ^225^Ac; four consecutive α-particle-emitting daughters are formed during decay (color figure online)

**Fig. 2:**
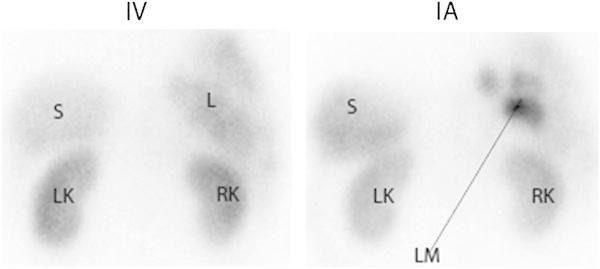
Planar posterior image of the liver 24 h after i.v. and 24 h after i.a. administration of ^111^In-octreotide. *LK* left kidney, *RK* right kidney, *S* spleen, *L* liver, *LM* three liver metastases visible after i.a. injection. After i.a. administration, there was increased tumor uptake of ^111^In-octreotide

A most promising recent development has been the application of α-particle-emitting radionuclides such as ^213^Bi or its mother radionuclide ^225^Ac (Figs. 1, 2) in PRRT. These radionuclides emit particles with a high energy (8.32 MeV for ^213^Bi/^213^Po and 27.5 MeV for ^225^Ac) combined with small particle ranges of only 50–80 μm. The LET is much higher for α particles than for β particles, which might further enhance the therapeutic efficacy of PRRT, especially in small tumor lesions including micro-metastases. Moreover, the cytotoxic effect of α radiation is independent of the cell cycle phase and oxygen concentration [46, 47], being beneficial especially for treatment of less oxygenated, hypoxic tumor regions. Moreover, the use of α-emitters minimizes the effect of cell cycle heterogeneity on tumor response to PRRT, whereas for β-emitters tumor responses do depend on cell cycle phase [48].

When α-emitters are stably complexed to targeting peptides and receptor density in normal tissue is relatively low, radiotoxicity in non-targeted normal tissues can be expected to be minimal, based on the short path length of α radiation. This was confirmed in a rat study in which ^213^Bi-DOTATOC showed a dose-related tumor anti-proliferative effect without side effects in normal organs [10]. In a pilot study in three patients, no short-term adverse side effects on kidney or bone marrow were found after ^213^Bi-DOTATOC, whereas there was a marked reduction in tumor vascularity and no progression of metastases during follow up for 9 months in patients with NET refractory to ^90^Y-DOTATOC or ^177^Lu-DOTATOC [49].

One of the safety concerns in relation to ^225^Ac is the formation of four consecutive daughter radionuclides during its decay. Safe application will be challenging, because the recoil kinetic energy delivered to the daughter nuclides during ^225^Ac decay is high, which might result in the presence of α-emitting daughters free from the targeting chelator–peptide complex. An accumulation of free α-emitters such as, for instance, ^213^Bi in the renal cortex may cause late nephrotoxicity as was shown at the highest doses used in mice studies with ^225^Ac-DOTATOC [47, 50]. A disadvantage of the use of ^213^Bi is its half-life of only 46 min and the fact that it is produced from a ^225^Ac generator that generates ^213^Bi for only 10–15 days. Nevertheless, if in phase I and II clinical trials, the use of α-emitters is shown to be safe, application of these radionuclides or a combination of α- and β-emitters might be a revolutionary way to target and eradicate tumors in NET patients.

## Intra-arterial administration

Unlimited growth of hepatic metastases resulting in liver failure is one of the most common causes of death in patients with gastroenteropancreatic NETs (GEP-NETs). This is why liver-directed therapies are developed, such as hepatic embolization of the liver metastases and debulking hepatectomy when indicated. In line with this approach, several research groups have examined whether local i.a. administration might, compared with systemic i.v. administration, increase uptake of radionuclides in hepatic metastases [51, 52]. Since hepatic metastases depend mainly on the hepatic artery for their supply of oxygen and nutrients, the higher arterial radiopeptide uptake during the first pass through the liver after i.a. administration was expected to lead to superior tumor uptake and better options for treatment of patients with a high metastatic liver load [52]. In a preclinical rat liver metastasis model, Pool et al. [53] demonstrated ^111^In-DTPA-octreotide tumor uptake to be twice as high after loco-regional administration via the hepatic artery than after i.v. administration. Furthermore, in a patient study, increased uptake of radionuclides in liver metastases has been reported after i.a. administration [54]. Kratochwil et al. [54] compared standard uptake values (SUVs) after i.a. administration of ^68^Ga-DOTATOC versus i.v. administration in 15 NET patients; SUVs were 3.75-fold higher after i.a. administration [54]. The same group [52] performed a pilot study in which ^90^Y- or ^177^Lu-DOTATOC was infused via the hepatic artery in 15 patients with liver metastases arising from GEP-NETs. This resulted in a higher rate of objective radiological responses than typically reported for the i.v. regime, i.e., 60 vs. 30 %, respectively. However, the promising observations of locally administered and β-particle-based PRRT need to be confirmed in a larger number of patients and compared with a proper control group treated intravenously.

In addition to the favorable higher uptake of radiolabeled SST analogs after i.a. administration, a locally higher serum concentration of the radiopeptide increases the risk of (partial) receptor saturation. Kratochwil et al. [52], using dynamic imaging, assessed pharmacokinetic data after i.a. and i.v. infusion of ^111^In-DOTATOC (250 MBq/150 μg) within the same patients (*n* = 4). They found i.a. administration to result in a 3.5-fold increased uptake in the initial phase, which decreased after 10 min, and according to the authors this was due to saturation effects. This indeed indicated that maximum achievable tumor uptake might be limited by receptor saturation. Therefore, a higher specific activity, which means an increased amount of radioactivity labeled to the same amount of SST analog, might be pivotal for this kind of therapy. Increased specific activity, either by optimization of the radiolabeling procedure or by labeling of the peptide with non-carrier-added ^177^Lu, might therefore allow enhanced levels of radionuclides within liver metastases after i.a. administration.

Even though i.a. administration is far more complex than i.v. administration, it has nevertheless been reported to be a safe procedure [54]. Therefore, considering the results achieved in pilot experiments, a randomized clinical trial comparing responses to PRRT after i.a. administration versus responses after i.v. administration in NET patients with a high hepatic tumor load would be of great interest, allowing a clear evaluation of the potential treatment benefits achieved after i.a. administration.

## Tumor dosimetry and organs at risk

### Organs at risk

Severe permanent renal toxicity (grade 4) has been observed to occur late (1–10 years) after the start of PRRT treatment with ^90^Y-DOTA-octreotide in 102 out of 1,109 (9 %) patients after a fixed activity of 3.7 GBq/m^2^ body surface area [55]. Severe hematological toxicity (grade 3–4) occurred in 13 % of the patients, mostly transient but in a few cases (3 out of 1,109) developing into myelodysplastic syndrome (MDS) or leukemia [44]. Hematological toxicity of equivalent severity (grade 3 or 4) was also reported in 10 % of 504 patients treated with ^177^Lu-DOTA-octreotate according to a fixed dosing scheme of 4 × 7.4 GBq, again with some (3 out of 504) developing into MDS 2–3 years after the last treatment [29]. The same level of hematological toxicity was reported by Sabet [56]: 23/203 (11 %) developed grade 3 and 4 hematological toxicity and three patients (1.4 %) developed MDS. Radiation-induced renal insufficiency has not been reported in any study of therapy with ^177^Lu-DOTA-octreotate alone.

### Kidney dosimetry

Physiological uptake of peptides in the kidneys is concentrated at the proximal tubuli distributed over the cortex where reabsorption of proteins from the primary urine back into the blood stream takes place. This uptake can be partially blocked by giving patients a co-infusion of amino acids, which results in 35 % reduction of renal uptake in PRRT performed in clinical practice [57]. Retrospective analysis of cases of late-occurring renal toxicity with ^90^Y-DOTA-octreotide showed that the absorbed dose is a predictor of renal toxicity [58]. Accurate dosimetry is needed, which accounts for both the individual kidney kinetics and the actual kidney volume irradiated. The absorbed dose to the kidneys per therapy cycle is also an important risk factor; a higher dose rate and a higher dose per fraction lead to more renal damage, as expressed by the BED, calculated according to the linear quadratic (LQ) model:

$$ {\text{BED}} = D\left( {1 + \frac{{T_{\mu } }}{{T_{\mu } + T_{\text{eff}} }} \times \frac{d}{{{\raise0.7ex\hbox{$\alpha $} \!\mathord{\left/ {\vphantom {\alpha \beta }}\right.\kern-0pt} \!\lower0.7ex\hbox{$\beta $}}}}} \right) $$

where *T*_*μ*_ is the repair half-life of repairable damage, *T*_eff_ the effective half-life of the kidney dose buildup, *α/β* the radiation sensitivity parameter, *d* the absorbed dose per therapy cycle, and *D* the total absorbed dose. The dose threshold for renal damage after external beam radiation given in 2 Gy fractions is 20–23 Gy, whereas after ^90^Y-DOTA-octreotide a 5–8 Gy higher threshold was observed, which could be well explained by the LQ model-based BED [59].

Renal toxicity by radiation exposure develops slowly after the initial tubular radiation damage. Besides the BED, additional risk factors are older age, diabetes, hypertension and use of nephrotoxic drugs prior to PRRT [60, 61]. From these findings, two absorbed dose thresholds are now being postulated: a BED of 40 Gy for patients without risk factors and a BED of 28 Gy for patients with multiple risk factors for renal problems [61]. Patients with risk factors also tend to show a higher dose to the kidneys per administered activity compared with patients without risk factors, although more patients than the 28 patients (including 11 with risk factors) studied by Guerriero et al. [62] are needed for significance.

Tailoring personalized PRRT to the absorbed dose limit requires dosimetric methods of the highest accuracy. The inter-patient variability in kidney dosimetry is too great to justify the use of a group-averaged absorbed dose, as is customary with diagnostic radiopharmaceuticals. The BED-based limits derived for ^90^Y-DOTA-octreotide therapy are assumed to be valid also for ^177^Lu-DOTA-octreotate, although no renal toxicity has been observed for this therapy. In a phase I trial with ^90^Y-DOTA-octreotide, a pure β-emitter, dosimetry was based on pre-therapeutic imaging with the PET analog ^86^Y-DOTA-octreotide [63]. This method resulted in proof of a correlation between BED and renal toxicity [58]. Kidney dosimetry for γ-emitters is traditionally based on planar imaging with activity quantification by the conjugate view method. In a comparison between conjugate view and quantitative SPECT imaging of the kidney uptake of ^177^Lu-DOTA-octreotate, the planar method resulted in an overestimation of the absorbed dose together with a high variance in background, both due to overlapping radioactivity [64]. Post-therapeutic planar imaging after PRRT with co-administration of ^111^In-DOTA-octreotide did, however, yield supporting evidence for the toxicity threshold to be used in PRRT with ^90^Y-DOTA-octreotide [59, 61].

In a dosimetry study in which 200 patients were treated with ^177^Lu-DOTA-octreotide the absorbed dose ranged between 2 and 10 Gy (median 4.5 Gy) per therapy cycle, corresponding to a BED range of 2–16 Gy (median 4.9 Gy) [65].

The difference in renal toxicity incidence after ^90^Y-DOTA-octreotide versus ^177^Lu-DOTA-octreotate at almost equivalent kidney doses seems to be evident. The radiation exposure by ^90^Y will be more homogeneous than by ^177^Lu, because of the longer tissue penetration range of the particles emitted by ^90^Y in comparison to the shorter range of those emitted by ^177^Lu. The activity distribution of the peptide in the kidney is not homogeneous as was shown in ex vivo autoradiographs of excised kidney sections from patients injected with ^111^In-DTPA-octreotide prior to nephrectomy [66]. The radioactivity was mostly confined to the cortex with a streaky pattern gradient from high concentration in the outer part to low concentration in the medulla [67]. Yttrium-90 resulted in a much more homogeneous dose distribution than ^177^Lu [78]. The absorbed dose distribution in the kidneys has also been calculated on SPECT/CT with ^111^In-DTPA-octreotide [68]. Uptake in the cortex and fall-off of the absorbed dose at the boundaries already introduce inhomogeneities in the dose distribution, although not as extreme as for ex vivo autoradiography-based ^177^Lu dose distribution. In the absence of clear dose-related renal toxicity, the exact dose limit for ^177^Lu-DOTA-octreotate is still unclear, and the sparing effect of the inhomogeneous dose distribution is also speculative.

### Bone marrow dosimetry

The absorbed dose to the bone marrow is not always routinely determined, as it involves regular blood sampling and determination of the whole-body distribution. The blood-based method is used for β-particle bone marrow dosimetry, given that, for peptides, the bone marrow radioactivity concentration is equivalent to the concentration in blood [69, 70]. The γ radiation from ^177^Lu gives an additional cross-dose from the total body and from organs and tumors with radioactivity uptake, which can form more than 60 % of the total bone marrow dose, but it also shows high variability [69]. The cumulative limit in absorbed bone marrow dose is considered to be 2 Gy, in line with the limits used for ^131^I thyroid cancer therapy [71] to prevent direct unrecoverable hematological toxicity. The probability for inducing leukemia and MDS, however, shows a linear relation with absorbed dose and it is unclear whether a dose limit would help to keep this risk within reasonable limits.

With standardized dosimetry methods no clear relationship has been reported between hematological toxicity and absorbed bone marrow dose [69, 72]. An almost linear relation is obtained between dose and reduction of platelet counts at nadir after ^90^Y-DOTA-octreotide therapy [69, 72]. The bone marrow dose needs to be corrected by a weight function aggravating the effects in patients with low baseline platelet counts without prior chemotherapy and normal recovery.

### Tumor dosimetry

The target for PRRT is metastasized disease including smaller and microscopic lesions, but it is difficult to determine the absorbed dose in lesions smaller than 1 cm in size. The absorbed dose needed for local control of pancreatic NETs with adjuvant external beam radiotherapy is in the order of 50 Gy [73]. With PRRT, the median absorbed dose to obtain a volume reduction of NETs by ^90^Y-DOTA-octreotide is 232 Gy [74]. The difference in doses can be partly explained by the difference in target size (tumor bed with minimal disease vs tumors ranging between 2 and 500 g) and the differences in dose rate and uniformity.

The absorbed dose to the tumor shows huge inter-patient variance. Liver metastases were reported to receive a dose of 167 ± 139 Gy for the first treatment cycle with 7.4 GBq [75]. Responders showed a >20 % decrease in absorbed dose in the following treatment cycles. Variance in the tumor dose and its reduction with each subsequent therapy cycle were also reported by Garkavij et al. [64]; the median absorbed dose to the tumor in their study of ^177^Lu-DOTA-octreotate-treated patient was reported to be 207 Gy (range 17–387 Gy).

### Treatment planning for PRRT

Hardly any centers follow a dosimetry-guided administration scheme for PRRT. Most PRRT therapies are given according to a fixed activity administration scheme. With ^90^Y-DOTA-octreotide the administered activity is scaled by the patient’s body surface area at doses of 3.7 GBq/m^2^. This dosing scheme is based on phase I trials with the compound, indicating a dose-limiting toxicity in the kidneys above 7.4 GBq/m^2^ after a short follow-up of 150 days and partial absence of kidney protection by amino acid infusions [76]. Longer follow-up of the patients in other phase I trials did show the benefit of dosimetry-guided therapy or, as a second option, of using lower administered activities per treatment cycle [58, 61, 63]. By lowering the activity per treatment cycle the total BED to the kidneys will decrease and thus reduce the risk of renal toxicity.

For ^177^Lu-DOTA-octreotate the most commonly used fixed dosing scheme is based on the protocol used by Kwekkeboom et al. [29]: four treatment cycles of 7.4 GBq. Originally some patients were excluded from getting the fourth treatment cycle, as they would otherwise have exceeded the conservative kidney dose limit of 23 Gy. This same limit of 23 Gy is applied in the dosimetry guided treatment schedule used by Sandström et al. [65]: the 200 patients in their study were treated by consecutive cycles of 7.4 GBq, until the 23 Gy limit was reached; 50 % of the patients received more than four cycles, with cycles received ranging between 2 and 10.

A treatment schedule based on dosimetry should focus on the absorbed dose both to the kidneys and to the bone marrow. Volume delineation of the renal cortex is not a straightforward procedure and it is time consuming when done manually. The exact volume is not needed when using average activity concentrations over a volume inside a representative sample of the kidney [65]. The quantitative SPECT method uses this same principle, but also transforms the activity concentration to an SUV by considering the total body uptake measured at 40-60 min after the ^177^Lu therapeutic dose before any voiding [77]. This same method is also used for determining the absorbed dose in a section of the patient’s spine as a representative sample for the bone marrow.

Patients who are retreated with two additional cycles of ^177^Lu-DOTA-octreotate PRRT after relapse following the first treatment do not show renal toxicity [78, 79]. Considering the variance observed in kidney dosimetry, a cumulative activity of 44 GBq could lead to a kidney BED of between 11 and 90 Gy, according to the range reported in 200 patients by Sandström et al. [65].

## Combination of PRRT with other treatments

Interesting combination options include PRRT as an adjuvant treatment after surgery, as this approach might prevent development of tumor lesions after spread of tumor cells during surgery, or eradicate micro-metastases that had already developed prior to surgery. This PRRT approach was studied in a preclinical model, mimicking perioperative tumor spill by injection of SSTR-positive tumor cells into the portal vein. In this study, ^177^Lu-DOTATATE treatments significantly reduced or prevented tumor development [80]. PRRT can also be applied as a neo-adjuvant treatment to achieve tumor size reduction and thus allow curative surgery. This was successfully performed recently in two patients with pancreatic NETs [81, 82].

Another option to improve anti-tumor response is to combine PRRT with chemotherapeutics; the latter may be applied to obtain radiosensitization of the tumor cells through modulation of cellular and molecular interactions causing, for example, enhanced DNA damage and repair, cell-cycle synchronization, apoptosis, tumor cell re-oxygenation or inhibition of cell proliferation. Radiosensitizing agents are commonly used in combination with external beam radiation therapy. Drugs with radiosensitizing effects based on cellular and molecular interactions include camptothecin, gemcitabine, and 5-fluorouracil (5-FU) or its prodrug capecitabine (Cap). The radiosensitizing effect of camptothecin is due to its ability to prevent DNA relegation by binding to topoisomerase I, which inhibits repair of single stranded breaks caused by radiation. Gemcitabine causes accumulation of tumor cells in the radiosensitive G2/M phase, making the tumor cells more sensitive to PRRT. Cap not only abrogates DNA replication through insertion of chain-stopping nuclides, it is also a thymidine synthetase inhibitor causing depletion of thymidine. Besides its radiosensitizing effects, Cap has also been described to deplete the tumor cell’s methylguanine DNA methyl transferase, an enzyme responsible for the repair of DNA damage caused by the DNA alkylating agent temozolomide (TMZ) [50]. In their clinical study, Claringbold et al. [83] used a treatment scheme based on these findings. This scheme consisted of 14 days of Cap treatment, starting 5 days before radiopeptide administration, and the administration of TMZ during the last 5 days of Cap treatment [83].

Until now the radiosensitizing effects as described above have been the main focus for clinical application of combinations of PRRT with chemotherapeutics [83–86]. Some challenges have to be faced during such studies though. Indeed, tumor uptake of radionuclides during PRRT depends on both tumor vascularization and SSTR expression, both of which can be affected by anticancer therapeutics [87–89].

In our preclinical study in mice, an increased tumor perfusion was measured for 14 days after TMZ treatment. This resulted in an increased uptake of radiopeptide after TMZ treatment [89].

Considering SSTR expression, Fueger et al. [87] examined the possible influence of cytotoxic or cytostatic agents on binding characteristics of an SST ligand in vitro. They found a reduced expression of high-affinity DOTA-LAN binding sites in response to incubation with gemcitabine, camptotecin, mitomycin C and doxorubicin (Table 1). In the case of gemcitabine, a four-day recovery eventually resulted in a significant up-regulation of SSTR. This was confirmed in a study by Nayak et al. [90], in which uptake of ^177^Lu-DOTATOC in cells in culture was 1.5–3 times increased 4 days after gemcitabine exposure compared with that in untreated control cells. Besides SSTR up-regulation the treated cells also showed cell cycle modulation; most of the viable cells were in the radiosensitive G2/M phase. These effects resulted in a synergistic effect of gemcitabine and ^177^Lu-DOTATOC [90].

**Table 1:** Combination of PRRT with other therapeutic agents

Therapeutic agent	Mechanism of action	Studies	Results	References
Gemcitabine	Chain stopper and ribonuclease reductase inhibitor Chromosome aberration Cell cycle synchronization	In vitro study	SSTR expression was downregulated during exposure, then restored and upregulated 4 days after exposure, resulting in synergism with ^177^Lu-DOTATATE	[87, 90]
Camptothecin	Binds to topoisomerase I and DNA complex, preventing DNA relegation Enhanced apoptosis Cell cycle arrest	In vitro study	SSTR expression was downregulated during camptothecin exposure	[87]
Mitomycin C	Cross-linking DNA	In vitro study	SSTR expression was downregulated during mitomycin C exposure	[87]
Cisplatin	Crosslinking DNA Repair inhibition	In vivo study	Cisplatin + ^177^Lu-DOTATOC was 23 % more effective than ^177^Lu-DOTATOC alone	[87]
Doxorubicin	Intercalating with DNA Cell cycle arrest	In vivo study	Doxorubicin + ^177^Lu-DOTATOC was 14 % more effective than ^177^Lu-DOTATOC alone	[87]
RAD001 (everolimus)	mTOR inhibitor Cell cycle arrest	In vivo study	RAD001 + ^177^Lu-DOTATATE was less effective than ^177^Lu-DOTATATE alone	[93]
5-fluorouracil or its prodrug capecitabine	Chain stopper and thymidine synthetase inhibitor Repair inhibition Cell cycle arrest	Phase II clinical trial	Combination with ^177^Lu-DOTATATE appeared to be safe	[84–86]
Temozolomide	DNA alkylating agent	Phase II clinical trial	Combination with capecitabine and^177^Lu-DOTATATE appeared to be safe	[83]

As RAD001 or everolimus has been shown to be effective against pancreatic NETs [91], a combination of PRRT with RAD001 could be another promising PRRT combination therapy option. In a preclinical study, however, the combination of RAD001 and PRRT was less effective compared with PRRT alone [68]. As RAD001 has been shown to cause a G1 arrest [92], Pool et al. [93] suggested this as a possible explanation for the reduced tumor response to the combination of mTOR-inhibitor everolimus (RAD001) with ^177^Lu-DOTATATE. Because NET cells have a peak of radio-resistance during the early G1 phase [48], the tumor cells may have been less sensitive to ^177^Lu-DOTATATE when administered after the start of RAD001 treatment.

As regards the clinical application of PRRT in combination with other anticancer agents, to date only phase II clinical trials have been reported. In these studies, PRRT using ^177^Lu-DOTATATE was combined with 5-FU or Cap, supplemented or not supplemented with TMZ. 5-FU combined with high-dose ^111^In-octreotide appeared to be safe in a study of 21 patients, but did not add to therapeutic response rates compared with ^111^In-octreotide alone [94]. Administration of 5-FU or Cap + PRRT was reported to be safe in the studies of Barber et al. [84] and van Essen et al. [85]. These authors decided to continue this study with a two-armed, randomized, prospective study to compare the combination of ^177^Lu-DOTATATE with Cap versus ^177^Lu-DOTATATE alone. Claringbold et al. [83, 86] concluded that both Cap and the combination of Cap and TMZ could be safely combined with PRRT. After their study combining Cap and TMZ with PRRT in 35 patients, the authors reported that response rates and progression-free survival times appeared to exceed results with ^177^Lu-DOTATATE as a single agent [83]. In their study, GEP-NETs showed better responses than enteric NETs. Therefore, the overall response rate seen in GEP-NETs almost certainly reflected the synergistic effect of TMZ, whereas the partial responses seen in enteric NET patients were attributable to the radiopeptide component of the multimodality therapy. This suggests that the optimal combination of PRRT and chemotherapy should be selected for each NET subtype.

### Concluding remarks

Even though PRRT is a most promising therapy for NET patients in whom curative surgery is no longer an option, there is still room for improvement as discussed in this review.

The increased tumor uptake of radionuclides reported in the case of radiolabeled SSTR antagonists or i.a. administration is promising. More clinical studies are now needed to establish the value of these approaches.

As regards the combined use of ^90^Y and ^177^Lu, there is a clear need for comparative studies before the effectiveness of this combination can be evaluated. Alpha-emitters have promising features; the first studies have just been performed and longer follow-up periods are now needed to investigate potential long-term toxicity.

With regard to individual dosimetry, a large percentage of patients might receive additional treatment cycles before reaching dose-limiting toxicity levels as has been determined in kidneys and bone marrow. However, whether or not additional cycles will have a major influence on tumor response is not yet known.

Combinations of PRRT with other anticancer therapies have appeared to be safe, but to date only phase II clinical trials have been reported. In addition, only a small number of anticancer agents have been combined with PRRT, leaving numerous possible options for further research.

In conclusion, several directions to improve PRRT effects have been indicated, but additional preclinical and especially translational and clinical research are needed to obtain further proof of value.
